# An improved method for automated calculation of the water‐equivalent diameter for estimating size‐specific dose in CT

**DOI:** 10.1002/acm2.13367

**Published:** 2021-07-22

**Authors:** Choirul Anam, Fahmi Rosydiansyah Mahdani, Winda Kusuma Dewi, Heri Sutanto, Pandji Triadyaksa, Freddy Haryanto, Geoff Dougherty

**Affiliations:** ^1^ Department of Physics Faculty of Sciences and Mathematics Diponegoro University Central Java Indonesia; ^2^ Department of Physics Faculty of Mathematics and Natural Sciences Bandung Institute of Technology Bandung West Java Indonesia; ^3^ Department of Applied Physics and Medical Imaging California State University Channel Islands Camarillo CA USA

**Keywords:** CT dose index (CTDI), patient dose, size‐specific dose estimates (SSDE), water‐equivalent diameter (D_w_)

## Abstract

**Purpose:**

The aim of this study is to propose an algorithm for the automated calculation of water‐equivalent diameter (D_w_) and size‐specific dose estimation (SSDE) from clinical computed tomography (CT) images containing one or more substantial body part.

**Methods:**

All CT datasets were retrospectively acquired by the Toshiba Aquilion 128 CT scanner. The proposed algorithm consisted of a contouring stage for the D_w_ calculation, carried out by taking the six largest objects in the cross‐sectional image of the patient's body, followed by the removal of the CT table depending on the center position (*y*‐axis) of each object. Validation of the proposed algorithm used images of patients who had undergone chest examination with both arms raised up, one arm placed down and both arms placed down, images of the pelvic region consisting of one substantial object, and images of the lower extremities consisting of two separated areas.

**Results:**

The proposed algorithm gave the same results for D_w_ and SSDE as the previous algorithm when images consisted of one substantial body part. However, when images consisted of more than one substantial body part, the new algorithm was able to detect all parts of the patient within the image. The D_w_ values from the proposed algorithm were 9.5%, 15.4%, and 39.6% greater than the previous algorithm for the chest region with one arm placed down, both arms placed down, and images with two legs, respectively. The SSDE values from the proposed algorithm were 8.2%, 11.2%, and 20.6% lower than the previous algorithm for the same images, respectively.

**Conclusions:**

We have presented an improved algorithm for automated calculation of D_w_ and SSDE. The proposed algorithm is more general and gives accurate results for both D_w_ and SSDE whether the CT images contain one or more than one substantial body part.

## 1 INTRODUCTION

With the continually increasing use of CT worldwide,^1^, ^2^ and with the concern regarding the probability of cancer occurrence in the future induced by relatively high radiation doses from CT, especially in pediatric patients,^3^, ^4^ an accurate estimation of CT dose is important. Nowadays, the most widely used metrics of CT dose are computed tomography dose index (CTDI) and dose length product (DLP).^5^ However, it has been shown that CTDI and DLP do not assess patient dose, but rather assess scanner output metrics independent of patient geometric‐size and tissue attenuation.^6^ They are both influenced by the input scanner settings such as tube current, tube voltage, rotation time, beamwidth, and pitch.^5^

The CTDI metric was initially proposed to improve the multiple scan average dose (MSAD) metrics for quantifying the CT scanner output dose.^7^ Subsequent modifications of CTDI such as CTDI_100_, CTDI_w_, and CTDI_vol_ were developed to facilitate the demand for CT dosimetry management.^8^, ^9^ Basically, CTDI measurement can be accomplished by integrating a single scan dose profile along the longitudinal axis (*z*‐axis), and then dividing it by the beam collimation. CTDI_100_, which is measured by a 100‐mm‐long pencil ionization chamber, raises questions about its efficiency inwide beam CT.^10^ This is because a length of 100 mm is deemed insufficient to accumulate the scattered dose distribution. The way to overcome this problem is to extend the phantom length to a minimum of 45 cm and to use a small volume ion chamber to reach dose equilibrium.^11^, ^12^

The CTDI_100_ varies across the field of view (FOV). In case of body CT imaging, for instance, the CTDI_100_ is typically a factor or two higher at the surface (CTDI_100,p_) than at the center (CTDI_100,c_). The average CTDI_100_ across the FOV is characterized by the weighted CTDI (CTDI_w_).^13^ To represent output dose involving a series of scans, it is important to take into account any overlaps or gaps between the x‐ray beams from consecutive rotations of the x‐ray source. This is characterized with volume CTDI (CTDI_vol_).^13^, ^14^ The CTDI_vol_ is measured using a standardized polymethyl methacrylate (PMMA) phantom, either with a fixed diameter of 16 cm to express the patient's head or a diameter of 32 cm and length of 15 cm to express the patient's body.^13^, ^14^ However, both phantoms do not reflect the diversity of patient size. In a clinical setting, the geometrical size of patients can run from less than 10 cm for a newborn child to more than 35 cm for an obese adult patient.^15^, ^16^ In addition, the PMMA phantom was specially designed to be homogenous, and does not reflect the heterogeneous nature of the human body. For these reasons, CTDI_vol_ reflects a proper and consistent index of scanner output only.

A new dose metric, the size‐specific dose estimate (SSDE), was introduced^17^ to fully accommodate the patient size and the scanner setting. Patient size was described by the effective diameter (D_eff_), which can be obtained from both the anterior posterior (AP) and lateral (LAT) dimensions. SSDE is calculated by normalizing CTDI_vol_ with a size conversion factor (*f*_size_) taken from AAPM 204.^17^ However, D_eff_ does not account for the attenuation effects of the body's constituent materials^18^ nor their respective densities,^19^ and therefore it is not accurate for dose estimation.

An improved, more accurate metric to include both patient size and tissue attenuation, the water‐equivalent diameter (D_w_) was proposed.^20^, ^21^ D_w_ can be calculated either from CT localizer radiographs or from axial CT images. The attenuation values from axial CT images can be calculated directly by averaging the CT number in a region of interest (ROI) containing that object, while calculation from CT localizer radiographs can be performed by properly calibrating the pixel values in terms of water attenuation.^22^ Daudelin et al.^23^ have evaluated the methods to quantify patient attenuation from CT localizer radiographs. Localizer‐based D_w_ calculation is highly affected by minification or magnification of the LAT dimension due to mis‐positioning of the patient from the iso‐center.^20^ Thus, measurement of D_w_ using reconstructed axial CT images provides a more accurate result.^20^

An accurate automated approach to finding D_w_, in order to obtain accurate patient dose, is in great demand.^24^, ^25^, ^26^, ^27^, ^28^, ^29^, ^30^ Anam et al.^25^ proposed a fully automated method to calculate D_w_ in a phantom and in human anatomic regions using a region of interest (ROI) automatically fitted to the patient border. The automated calculation produced an excellent correlation to the manual calculation (*R* ^2^ = 0.999). Özsoykal et al.^26^ designed a patient contour upon the exclusion of irrelevant objects such as the CT table or clothes from the original image. The threshold value was then determined via trial and error until a complete segmentation of the body contour was achieved. Gharbi et al.^27^ also successfully proposed an automated method to measure D_w_ and SSDE based on the Fuzzy C‐means classification algorithm and edge detection. Recently, Juszczyk et al.^24^ proposed an automated segmentation of patient images to calculate D_w_ and SSDE using a convolutional neural network (CNN) and reported accurate results. The algorithms were effectively designed to calculate D_w_ based on segmentation of the largest object.^28^ Previous algorithms failed to segment the patient body in the case of images which consisted of more than one substantial separated body part, for example, chest images when the patient's arm was within the image and separated from the chest, and the lower extremities images where the two legs are separated. This inaccuracy of patient image segmentation led to an underestimate of D_w_ and an overestimate of SSDE. Therefore, we propose an improvement of the automated D_w_ calculation which will accurately measure SSDE even in thoracic examinations which include the patient's arms and lower extremities examinations which include separated legs. Our algorithm is expected to result in a general and more accurate patient segmentation from images whether they consist of one or more substantial separated objects. Thus, the estimate of patient doses (SSDE) will be more accurate and robust, so that the dose optimization through the diagnostic reference level (DRL) on the basis of SSDE can be increased.

## 2 METHODS

### 2.1 Images

**FIGURE 1:**
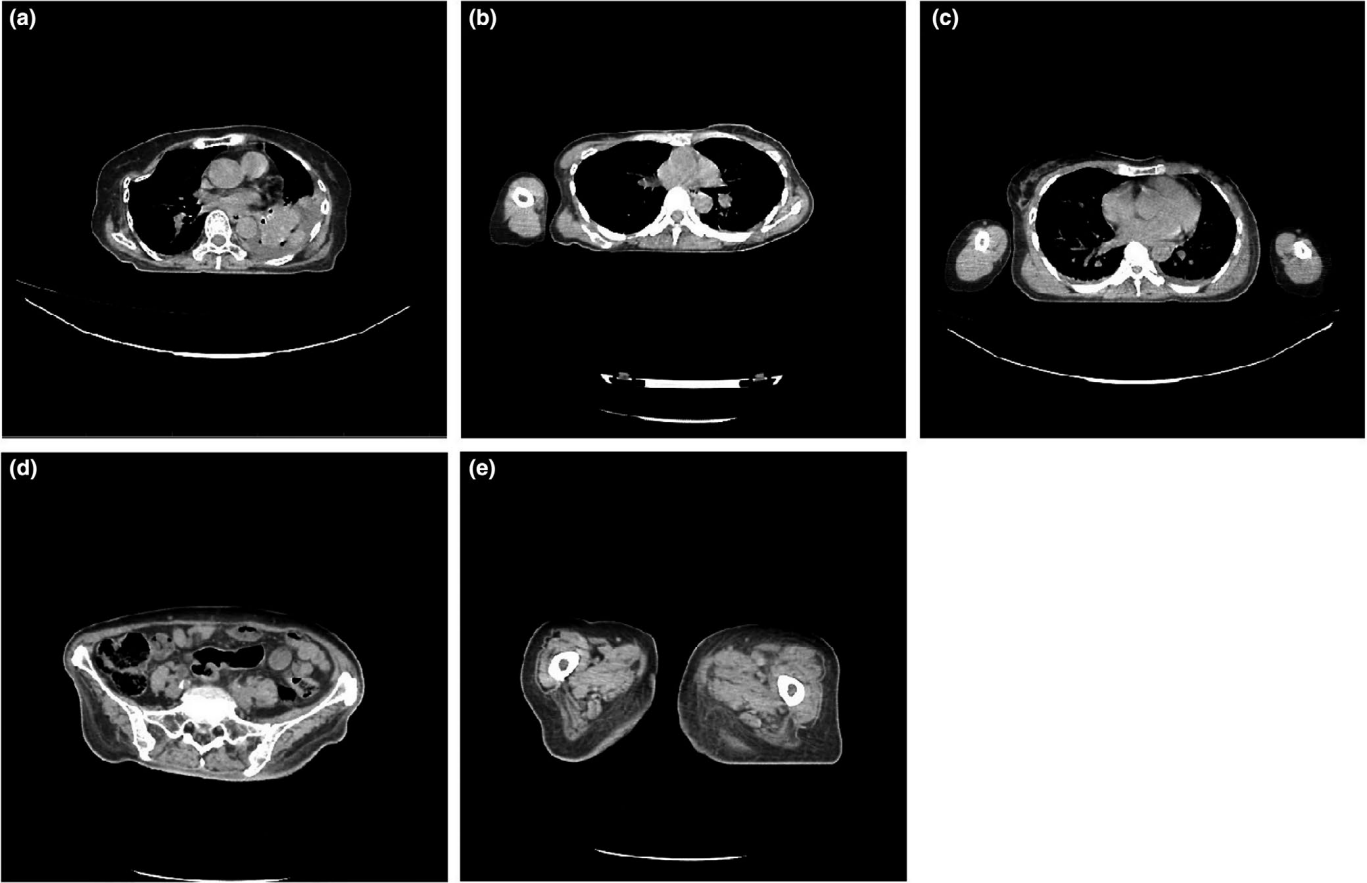
Examples of various axial images used in this study. (a) chest region with both arms raised up, (b) chest region with one arm placed downwards, (c) chest region with both arms placed downwards, (d) pelvic region consisting of one substantial object, and (e) lower extremities (legs) consisting two separated areas. Images (a) and (d) show one substantial object, while images (b), (c), and (e) show more than one substantial separated objects

This was a retrospective study on datasets from a hospital where anonymization and confidentiality were performed. All datasets were obtained from a multi‐detector computed tomography (MDCT), the Toshiba Aquilion 128, between August 2014 and December 2014. The dataset consisted of axial images of adult patients who had undergone chest, pelvic, and lower extremities CT examinations. The chest datasets were classified into three groups: 40 datasets with both arms raised (Figure 1a), 40 datasets with one arm placed downwards (Figure 1b), and 20 datasets with both arms placed downwards (Figure 1c). The pelvic (one substantial object [Figure 1d]) and lower extremities (with two separated legs [Figure 1e]) each consisted of 38 datasets.

### 2.2 Proposed algorithm

**FIGURE 2:**
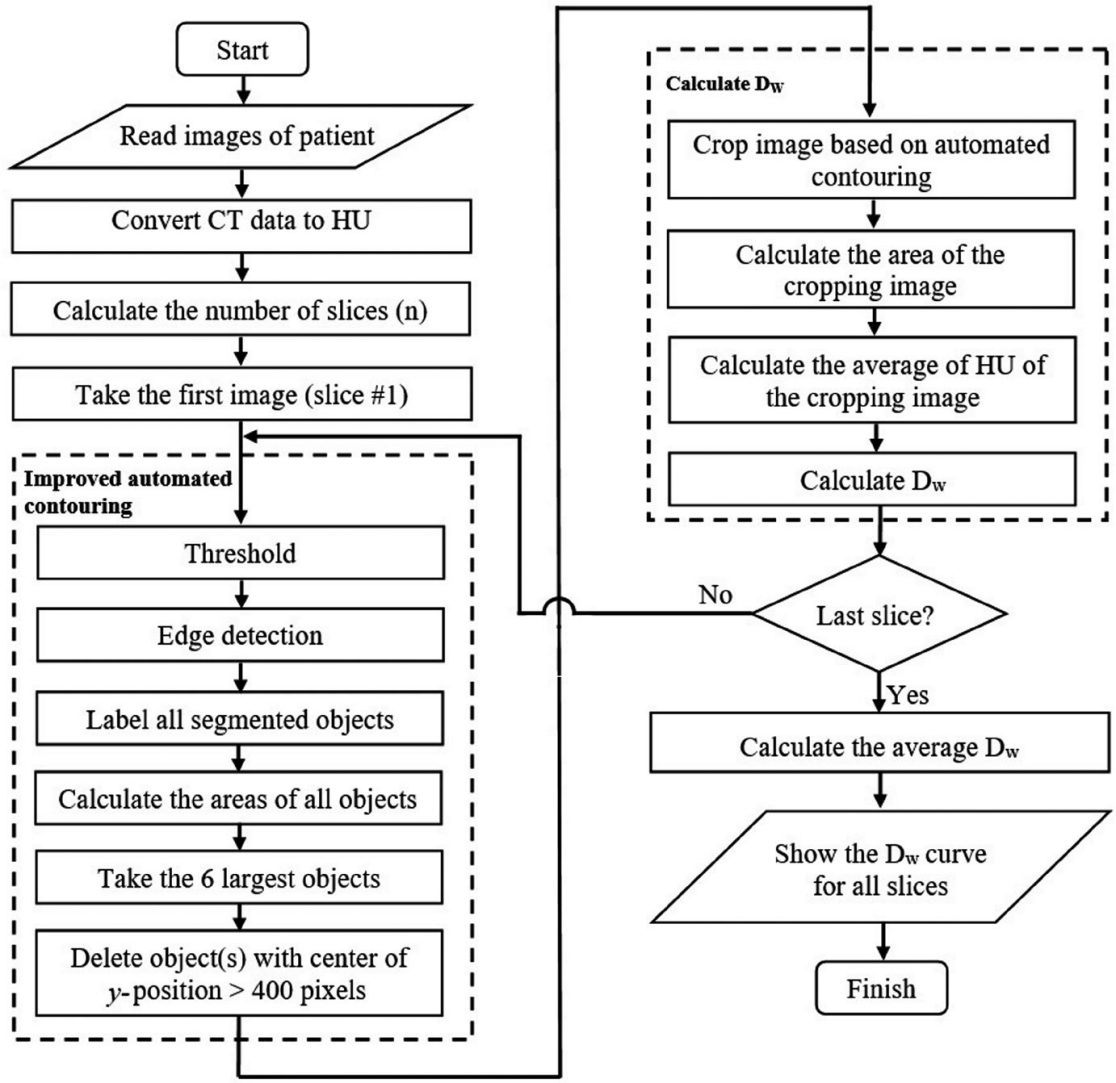
Flowchart of the proposed algorithm

The flowchart of the proposed algorithm is presented in Figure 2. The current method is a modification of our previous algorithm.^25^ The previous automated D_w_ calculation employed a threshold operation using a HU of −200, edge detection, and setting the largest object as the patient boundary.^25^ It agreed well (to within <0.5%) with the manual calculation.

For images involving multiple parts of the patient's body, the contouring process based on the largest object produces an inaccurate estimate of D_w_. Therefore, instead of using the largest body part as the patient border, the current method uses the six largest body parts. If the image consists of more than one substantial body part, the algorithm successfully detects the edges of all these body parts. On the other hand, if the image consists of only one substantial body part, then the five smaller detected body parts located within the one largest body part will be united into one large body part.

**FIGURE 3:**
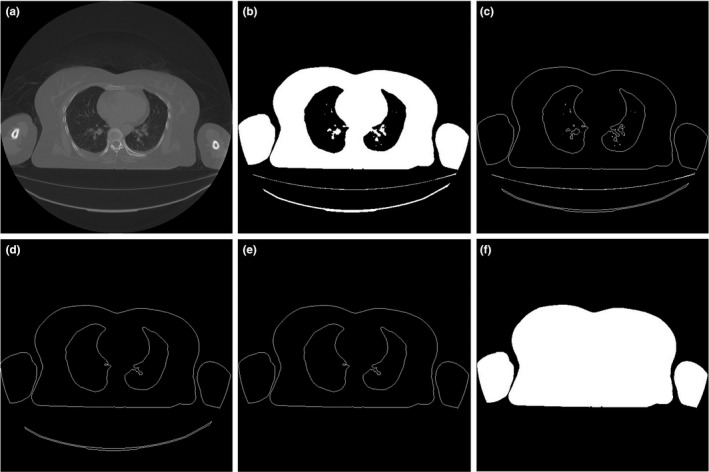
Proposed automated contouring stages: (a) initial image after converting CT data into HU, (b) binary image as a result of thresholding, (c) result of edge detection, (d) image showing the 6 largest objects including the patient table, (e) patient boundary after table removal, and (f) result of automated contouring after assigning all pixels within patient boundary with a value of 1

The stages of the automatic contouring are shown in Figure 3. A threshold operation was applied to the patient image (Figure 3b) after converting the DICOM image into HU. The perimeter of the patient was identified using edge detection (Figure 3c) and each object was labeled. The areas of all the objects were calculated, and the six largest objects were identified (Figure 3d). The patient table, however, is often detected at this stage.^18^, ^31^ To remove it, we determined the center positions (*y*‐axis) of the six objects and eliminated the objects whose positions are larger than 400 pixels from the top of the image (Figure 3e). This is based on the fact that the patient table is located at the bottom and is usually located at a position of more than 400 pixels, although a previous study has shown that a limit of 450 is sufficient to detect a patient table in many conditions.^32^ The last stage of the automated contouring was to assign all pixel within patient boundary with a value of one (Figure 3f).

The area and the mean HU of the image within the automated ROI result (i.e., patient boundary) were then calculated. The value of D_w_ was determined using the following equation:

(1) $$D_{w}=2\sqrt{\left[\frac{1}{1000}\bar{\text{HU}{\left(x,y\right)}_{\text{ROI}}}+1\right]\frac{A_{\text{ROI}}}{\pi }}$$

where A_ROI_ describes the patient area in the cropped image and $\text{HU}{\left(x,y\right)}_{\text{ROI}}$ is the mean HU contained in the ROI. The D_w_ calculation was performed for all the slices in the scanning range and an average value of D_w_ was calculated.

### 2.3 SSDE values

The value of SSDE can be determined by normalizing the CTDI_vol_ extracted from the DICOM information of the patient with the size conversion factor ($f_{\text{size}}$) based on Dw obtained from AAPM 204^17^ and AAPM 2020.^20^ SSDE value was calculated from the following equation:

(2) $$\text{SSDE}={\text{CTDI}}_{\text{vol}}\times f_{\text{size}}$$

**FIGURE 4:**
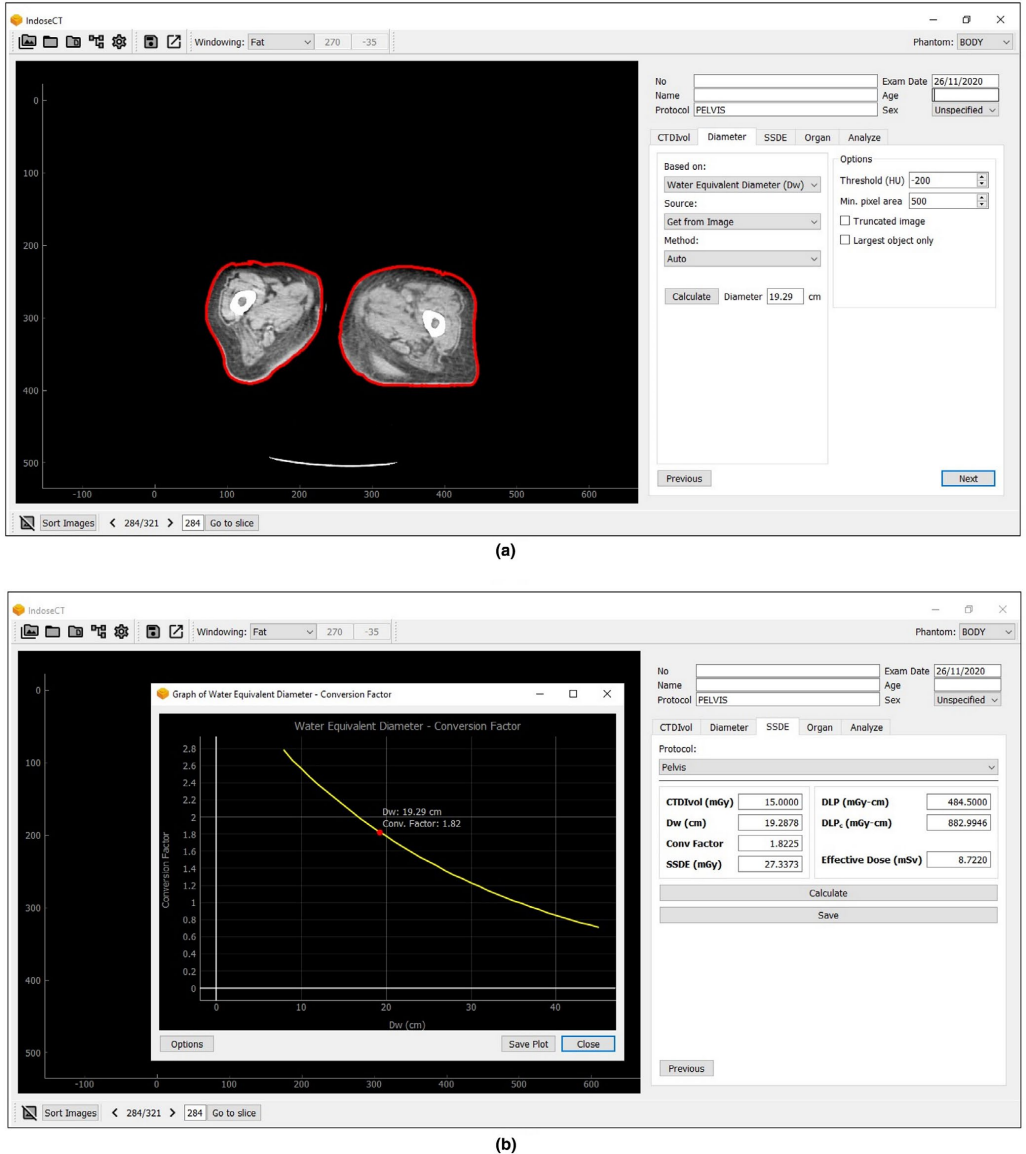
The screen displays of the graphical user interface (GUI) of IndoseCT showing (a) D_w_ calculation and (b) SSDE calculation

The outcomes of these calculations (denoted as D_w,n_ to define water‐equivalent value based on the new algorithm and SSDEn to represent patient dose estimation from the new algorithm) were correlated with the results measured by the previous algorithm (old algorithm) (D_w,o_ and SSDE_o_). Both algorithms were effectively integrated into a simple graphical user interface (GUI) of IndoseCT (i.e., an in‐house software for measuring SSDE) as depicted in Figure 4. The differences between D_w,n_ and D_w,o_ were calculated using Equation (3) and the differences between SSDE_n_ and SSDE_o_ were calculated using Equation (4).

(3) $$\Delta D_{w}\left(\%\right)=\left(\frac{D_{w,n}‐D_{w,o}}{D_{w,o}}\right)\times 100\%$$

(4) $$\Delta \text{SSDE}\left(\%\right)=\left(\frac{{\text{SSDE}}_{n}‐{\text{SSDE}}_{o}}{{\text{SSDE}}_{o}}\right)\times 100\%$$

## 3 RESULTS

**FIGURE 5:**
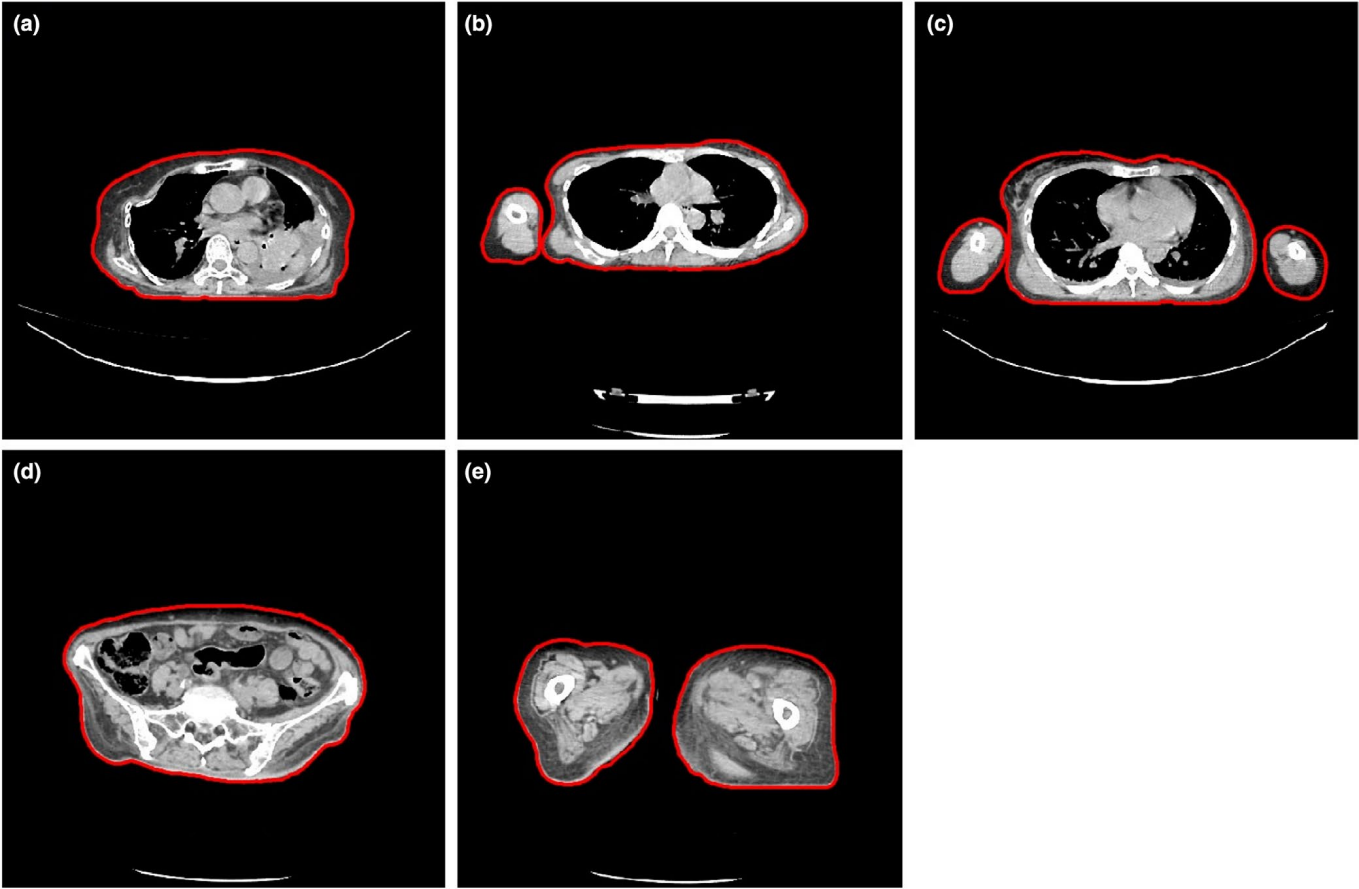
Images showing results of contouring based on the new improved algorithm. It shows that all of the patient's body in the image can be fully contoured whether the images containing one or more than one substantial separated objects

**FIGURE 6:**
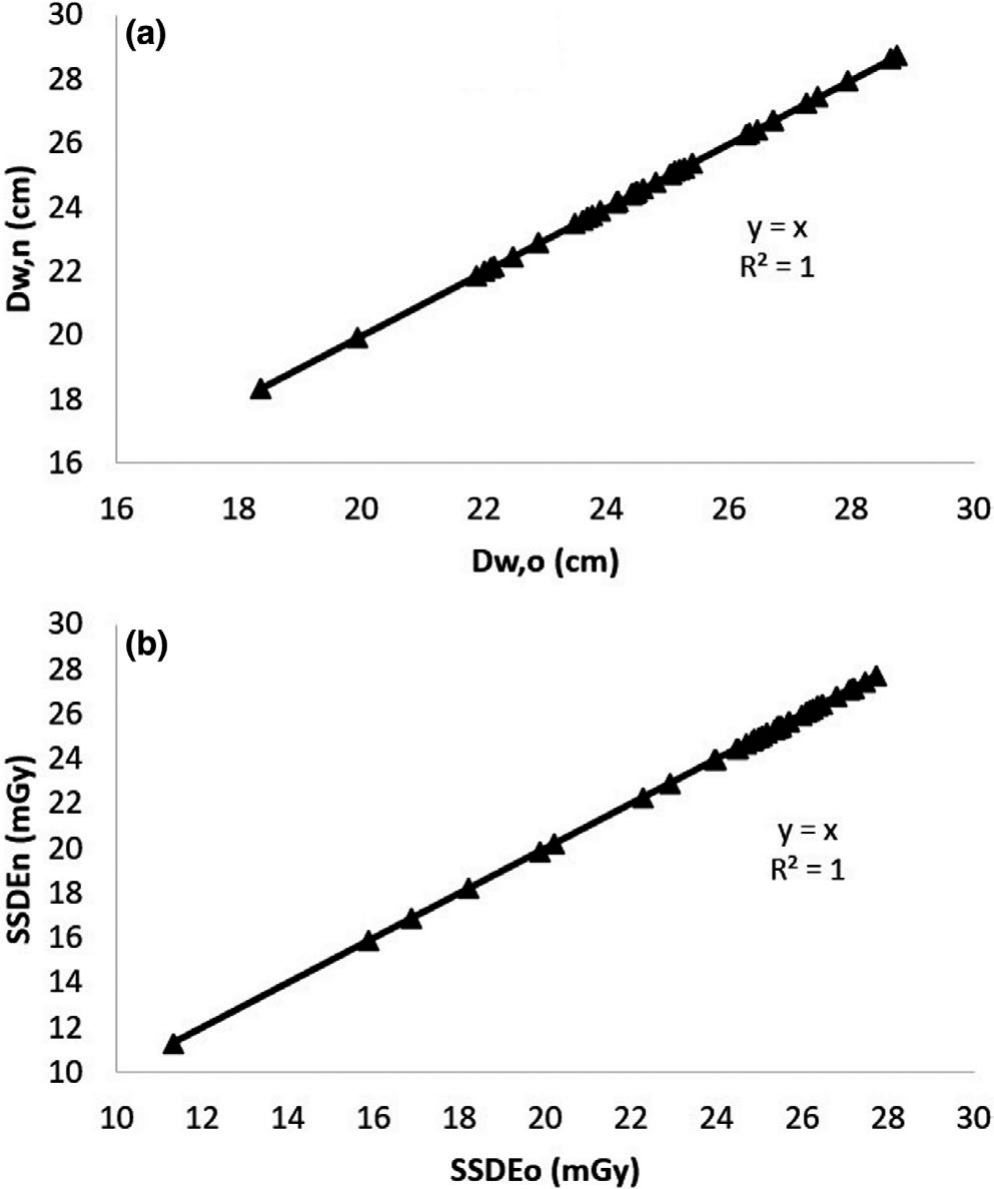
Correlations between the values of D_w_ (a) and SSDE (b) calculated based on the old algorithm (D_w,o_ and SSDE_o_) and the new algorithm (D_w,n_ and SSDE_n_) in the chest images with both arms raised up

**FIGURE 7:**
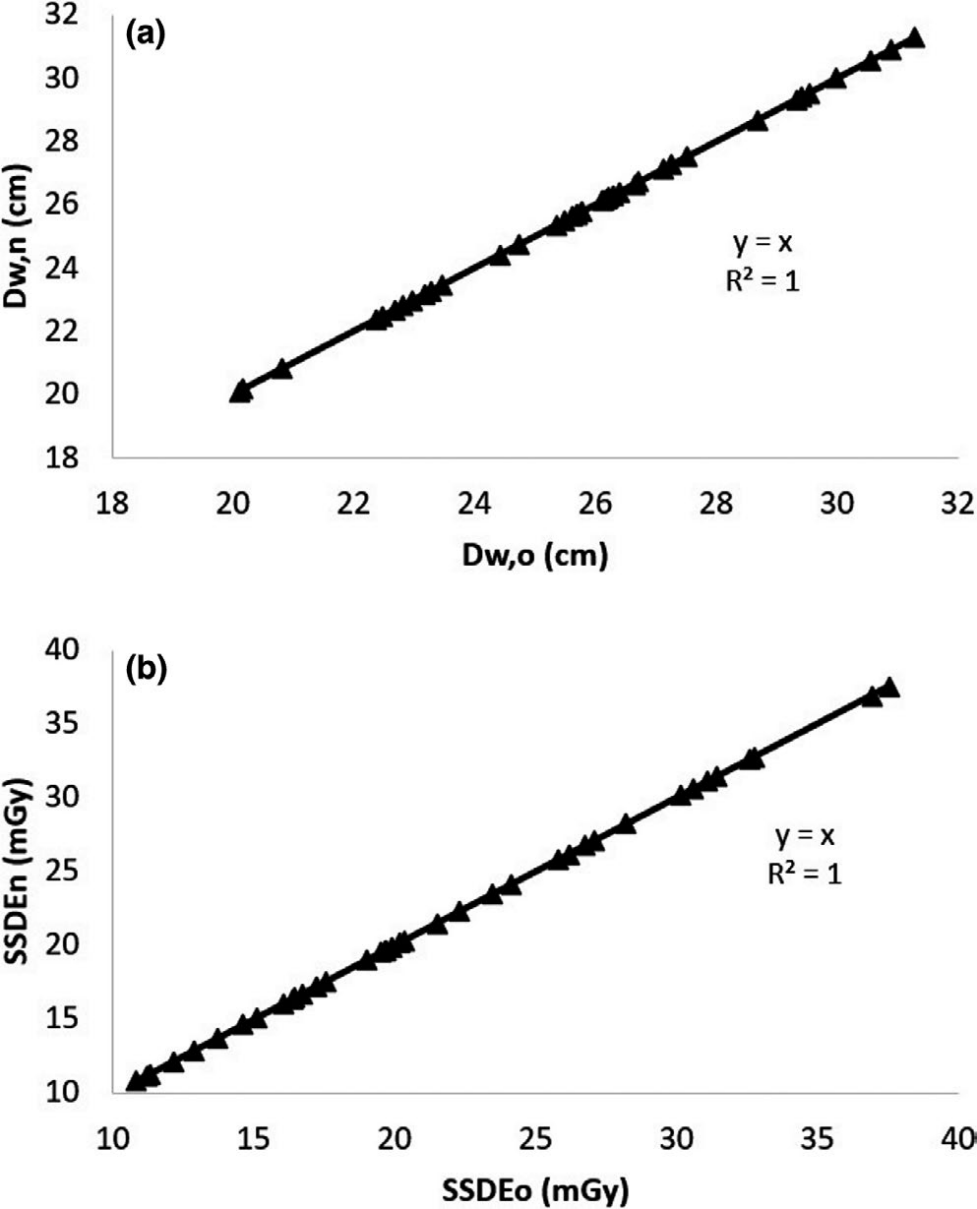
Correlations between the values of D_w_ (a) and SSDE (b) calculated based on the old algorithm (D_w,o_ and SSDE_o_) and the new algorithm (D_w,n_ and SSDE_n_) in the pelvic images

Figure 5 shows the result of our improved algorithm. All of the patient's body appearing in the image can be fully detected. The correlations of D_w_ and SSDE based on the previous and the new algorithms for the chest region with both arms raised up are presented in Figure 6, and for the pelvic region in Figure 7. The coefficients of linear correlation (*R* ^2^) for both regions are equal to one.

The results of D_w_ and SSDE based on the old and the new algorithms for images consisting of one substantial object are tabulated in Table 1. The two algorithms produce exactly the same values of D_w_ and SSDE, as expected.

**TABLE 1:** Results of Dw and SSDE based on the old^25^ and new algorithms and their differences for various axial images consisting of one substantial object

The cross‐sectional image	Water‐equivalent diameter (cm)		Percentage different (%)	SSDE (mGy)		Percentage different (%)
	D_w,o_	D_w,n_		SSDE_o_	SSDE_n_	
Chest + both arms raise up	24.56 ± 2.15	24.56 ± 2.15	0	24.24 ± 3.46	24.24 ± 3.46	0
Pelvic	25.77 ± 2.91	25.77 ± 2.91	0	21.81 ± 7.42	21.81 ± 7.42	0

**FIGURE 8:**
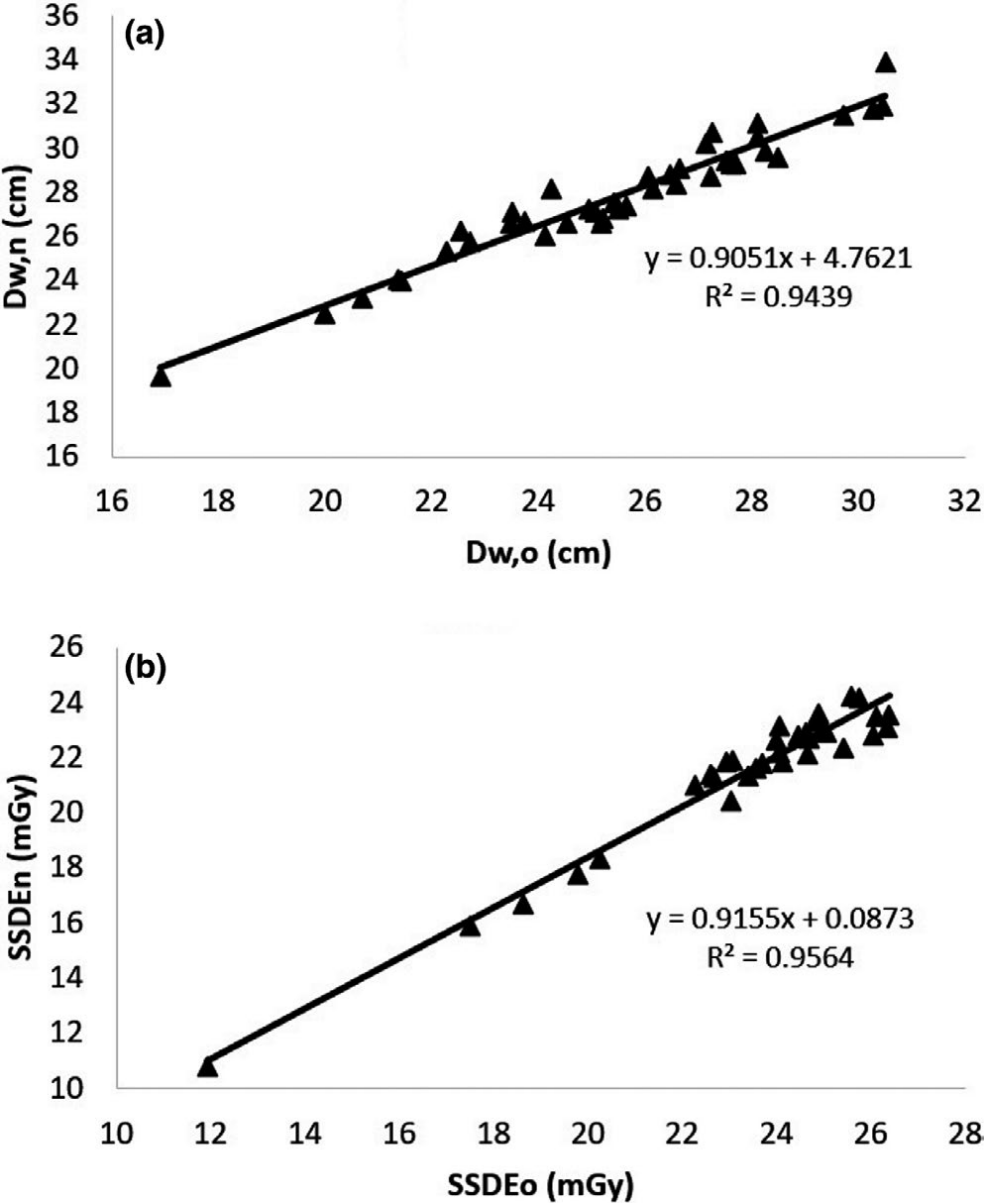
Correlation between the values of (a) D_w_ and (b) SSDE from the old and the new algorithm in the chest images with one arm down

**FIGURE 9:**
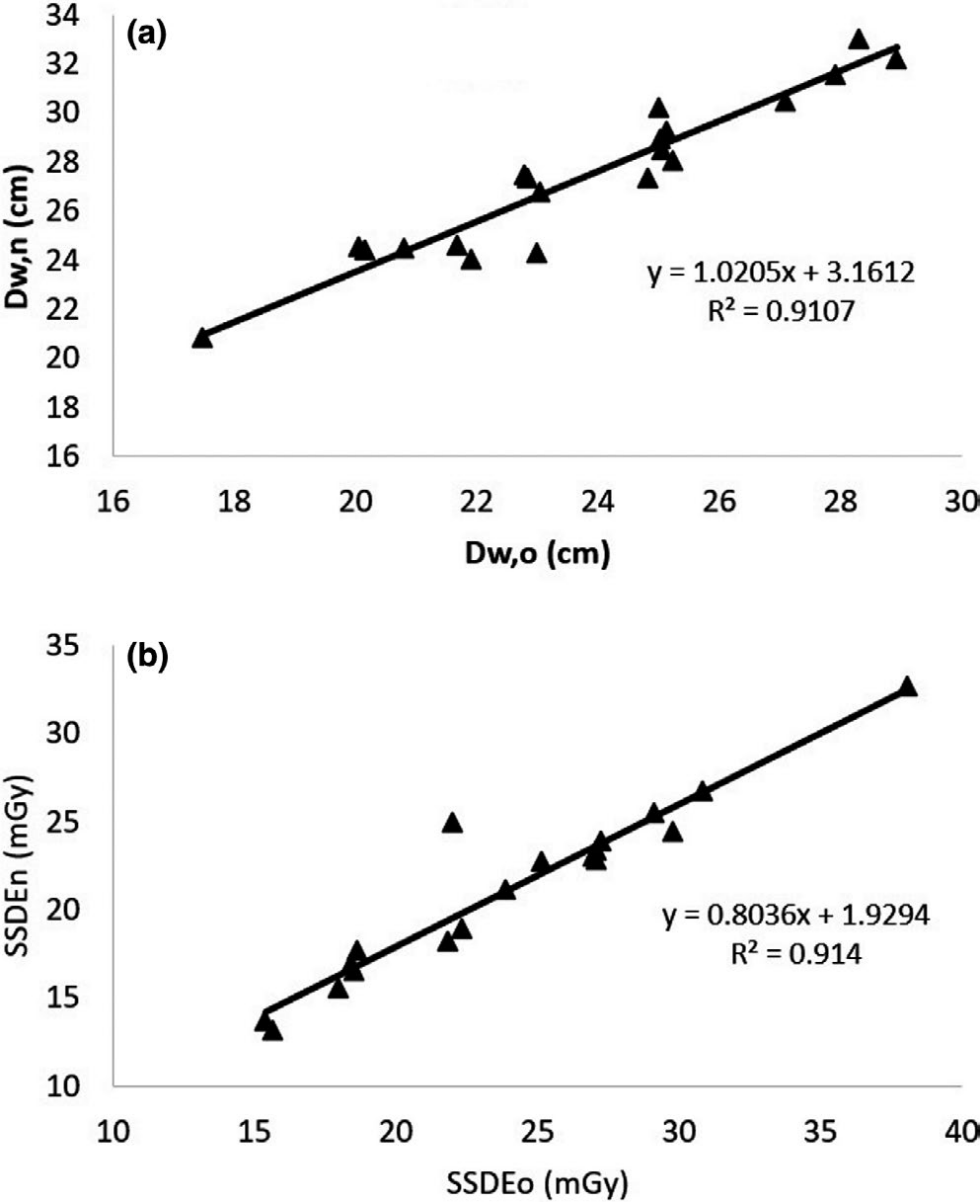
Correlation between the values of (a) D_w_ and (b) SSDE from the old and the new algorithm in the chest images with both arms down

**FIGURE 10:**
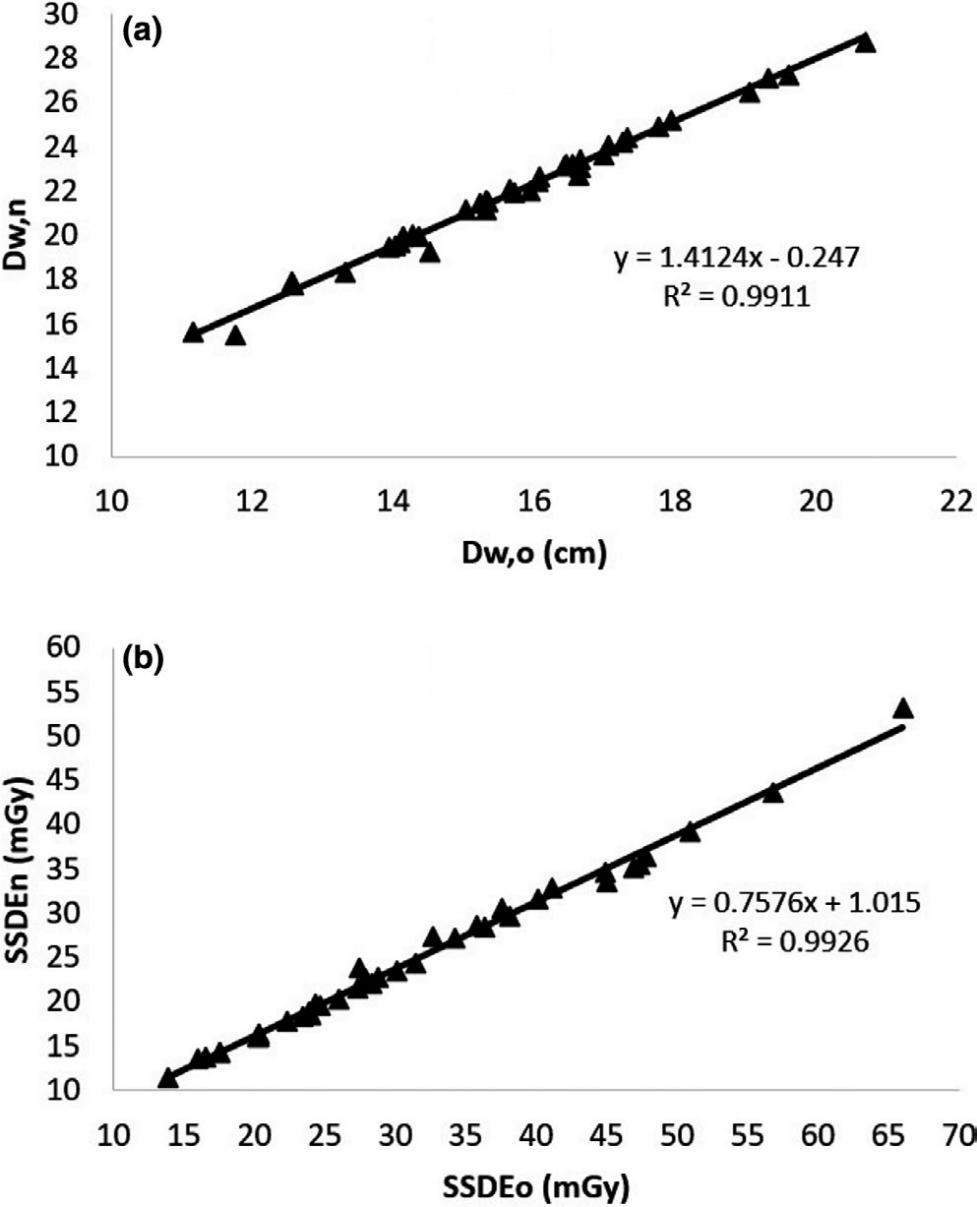
Correlation between the values of (a) D_w_ and (b) SSDE from the old and the new algorithm in the images containing two separated legs

It is interesting to analyze the results of D_w_ and SSDE based on the old and the new algorithms for images consisting of more than one substantial object. The correlations of D_w_ and SSDE values from the old and the new algorithms for the chest region with one arm down are presented in Figure 8 (*R* ^2^ > 0.94 for both D_w_ and SSDE), for the chest region with both arms down in Figure 9 (*R* ^2^ > 0.91 for both D_w_ and SSDE), and for separated leg images in Figure 10 (*R* ^2^ > 0.99 for both D_w_ and SSDE).

The differences of the results of D_w_ and SSDE based on the old and the new algorithms for images consisting of more than one substantial object are tabulated in Table 2. The percentage differences were averages for each dataset. The differences in the chest region with two arms down are larger than with one arm down, as predicted. Since the areas of two separated legs are comparable, the differences of D_w_ with the old and new algorithms is significant, that is, about 40%. It clearly shows that the calculation of D_w_ based on the old algorithm for images consisting of more than one substantial object gives lower D_w_ values than those from the new algorithm. This leads to the SSDE values calculated by the old algorithm being greater than those calculated by the new algorithm.

**TABLE 2:** Results of D_w_ and SSDE based the old^25^ and new algorithms and their differences for various axial images consisting of more than one substantial object

The cross‐sectional image	Water‐equivalent diameter (cm)		Percentage difference (%)	SSDE (mGy)		Percentage difference (%)
	D_w,o_	D_w,n_		SSDE_o_	SSDE_n_	
Chest + 1 arm down	25.47 ± 3.00	27.81 ± 2.79	9.49 ± 3.52	23.38 ± 2.99	21.47 ± 2.78	8.19 ± 2.38
Chest + 2 arms down	23.80 ± 3.00	27.45 ± 3.21	15.35 ± 4.57	23.98 ± 5.90	21.20 ± 4.96	11.19 ± 6.81
Two legs	15.75 ± 2.12	22.00 ± 3.00	39.64 ± 2.06	31.92 ± 12.32	25.20 ± 9.37	20.55 ± 2.72

## 4 DISCUSSION

Care should be taken in defining patient boundaries for automated calculation of D_w_. The auto contouring must be able to involve all parts of the patient's body and minimize the contribution of irrelevant objects such as the CT table. Setting the patient boundaries based on the largest object area (i.e., old algorithm^25^, ^27^) is not sufficient for calculating D_w_ accurately in many clinical cases.

The inaccuracy of the old algorithm for D_w_, as well as SSDE, is particularly noticeable when contouring images involving multiple body parts, which usually occurs in a clinical setting such as in an emergency condition or in radiotherapy. In an emergency CT scan, for example, traumatic patients cannot raise their arms over the heads due to trauma injuries, and have to place their arms by their side. Kweon et al.^33^ showed that placing arms at the side of the body gives a higher CTDI_vol_ and DLP. For this reason, we modified our previous algorithm to determine the patient contour from the six largest objects so that the entire patient body, including separated arms or legs, can be included.

When images consist of more than one substantial object, such as chest images with arms of the patient or images with two separated legs, the new algorithm is able to detect all parts of the patient within the image. Based on the new contouring, the new algorithm gives significantly higher D_w_ values and lower SSDE values compared to the older algorithm. The D_w_ results from the new algorithm were 9.5%, 15.4%, and 39.6% higher than those from the previous algorithm for chest images with one arm placed down, both arms placed down, and images with two separated legs, respectively. The SSDE results from the new algorithm were 8.2%, 11.2%, and 20.6% lower than those from the previous algorithm for the same cases.

The CT table may be among the six largest objects in the field of view. Previous studies have reported the impact of the CT table on the CT dose.^31^, ^34^ Li et al.^31^ found that almost 12% of the total attenuation in small‐sized objects comes from the presence of a CT table. Anam et al.^34^ showed that the calculation of D_w_ with the table included in the ROI gave a higher value by 1.5%–6.2%, and the SSDE is smaller by 1.0%–5.5%, than the calculations without consideration of the patient table. In this current study, we completely excluded the attenuation contribution from the CT table, based on its position (average *y*‐position >400 pixel). However, the position of the table can vary based on many factors, such as field of view and size of the patient. Investigation of the limit of *y*‐position for more clinical datasets from different scanners and various field of views will be useful.

In addition, in the current study, the threshold value for segmentation is adopted from a previous study (i.e., −200 HU)^25^ without any further evaluation. Although the previous study reported that this threshold value gives accurate results for the AAPM phantom, the accuracy of the threshold value for clinical images has not been established. A specific clinical image may require different threshold values to produce more accurate segmentation results. This needs a comprehensive further study. In the current study, the automated segmentation results were not compared with those of radiologist experts, so the two segmentation results were analyzed using evaluation metrics such as dice coefficients.

Detection of the six largest objects and removal of the patient table results in a more accurate measure of D_w_ and SSDE for all images. If the earlier algorithm was used for measuring the D_w_ and SSDE from images with more than one substantial object, the results can be corrected using the relationships in Figures 8, 9, 10 (*R* ^2^ > 0.9). The new modified algorithm is a fast and simple alternative way of providing accurate values of patient diameter and estimated dose.

## 5 CONCLUSIONS

An improved automated method to calculate D_w_ and SSDE estimates is presented in this study. The algorithm employs detection of the six largest objects within an image and deletion of the CT table. The algorithm gives the same results for D_w_ and SSDE as the earlier algorithm when images consist of only one substantial object. However, with images consisting of more than one substantial object, the new algorithm is able to detect all parts of a patient within the image and subsequently gives significantly higher D_w_ values and lower SSDE values compared to the earlier algorithm. The percentage differences of the D_w_ values between the new and old algorithms are 9.5%, 15.4%, and 39.6% for the chest region with one arm placed down, both arms placed down, and images with two separated legs, respectively. The new algorithm represents a more general approach for accurate D_w_ and SSDE estimation.

## CONFLICT OF INTEREST

Dr. Choirul Anam and Dr. Freddy Haryanto are IndoseCT developers. This software is not commercially available. It can be made available on request for research purposes. Rest of the authors have no conflicts of interest to disclose.

## AUTHOR CONTRIBUTION

CA and FH conceived the idea. CA and FRM developed the software. WKD, HS, PT, and CA calculated the D_w_ and SSDE on patient images based on the developed software. WKS, CA, and GD wrote the paper. All authors approved the final version of the paper.
